# Supplementary material for: Hepatitis B virus promotes liver cancer by modulating the immune response to environmental carcinogens
Source: Nat Commun. 2025 Jun 27;16:5360. doi: 10.1038/s41467-025-60894-z (PMC12205058; doi:10.1038/s41467-025-60894-z)
Supplement: Supplementary file 1 — Supplementary Information [file 41467_2025_60894_MOESM1_ESM.pdf]

Supplementary Information

Hepatitis B virus promotes liver cancer by modulating the immune response to environmental carcinogens

Mei Huang[1,2,3,8], Dongyao Wang[1,2,4,8], Jiao Huang[1,2], An-Na Bae[5], Yun Xia[1,2], Xutu Zhao[1,2], Mahsa Mortaja[1,2], Marjan Azin[1,2], Michael R. Collier[6], Yevgeniy R. Semenov[6,7], Jong Ho Park[1,2,5]*, and Shadmehr Demehri[1,2,9]*

[1]Center for Cancer Immunology, Krantz Family Center for Cancer Research, Massachusetts General Hospital and Harvard Medical School, Boston, MA, USA
[2]Cutaneous Biology Research Center, Department of Dermatology, Massachusetts General Hospital and Harvard Medical School, Boston, MA, USA
[3]Department of General Surgery, The First Affiliated Hospital of USTC, Division of Life Sciences and Medicine, University of Science and Technology of China, Hefei, China
[4]Department of Hematology, The First Affiliated Hospital of USTC, Division of Life Sciences and Medicine, University of Science and Technology of China, Hefei, China
[5]Department of Anatomy, School of Medicine, Keimyung University, Daegu, South Korea.
[6]Department of Dermatology, Massachusetts General Hospital and Harvard Medical School. Boston, MA, USA.
[7]Laboratory of Systems Pharmacology, Harvard Program in Therapeutic Science, Harvard Medical School, Boston, USA.
[8]These authors contributed equally
[9]Lead author

***** Authors for correspondence: jpark@dsmc.or.kr (JHP), sdemehri1@mgh.harvard.edu (SD)

Supplementary Figures

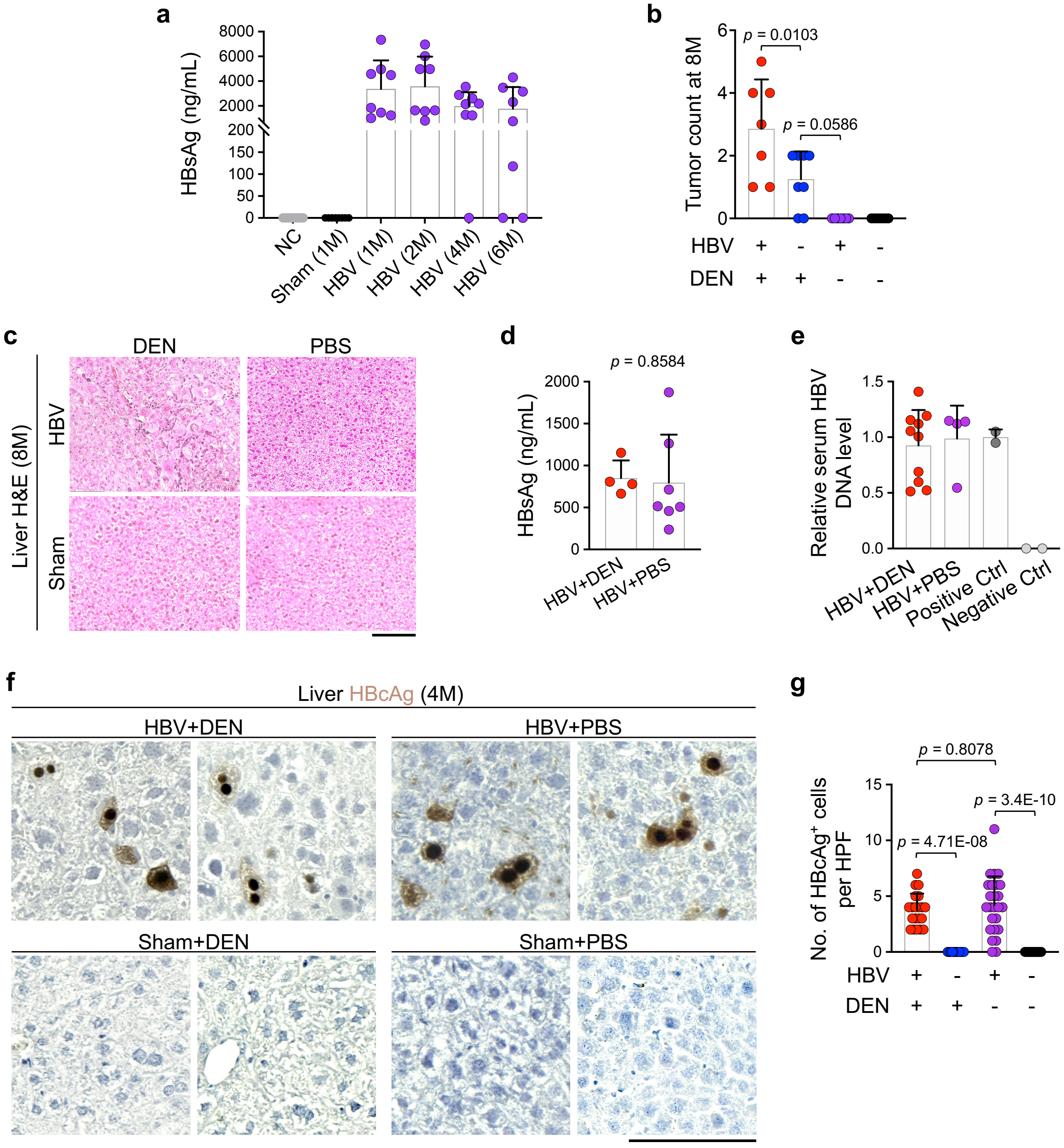

Supplementary Fig. 1, related to Fig. 1.

Persistent liver infection after pAAV-HBV hydrodynamic injection and liver cancer induced by HBV+DEN treatment. a Serum HBsAg levels in WT mice at 1, 2, 4, and 6 months after hydrodynamic injection of pAAV-HBV plasmid. NC, negative control; pAAV-sham was used as Sham control (n = 8 mice per group). Each dot represents a mouse. **b** Liver tumor counts in WT

mice that underwent liver carcinogenesis protocol at 8 months post-infection. n = 7 mice in HBV+DEN group, n = 8 mice in Sham+DEN group, n = 7 mice in HBV+PBS group, and n = 7 mice in Sham+PBS group. Each dot represents a mouse. **c** Representative images of H&E stained liver from WT mice that underwent liver carcinogenesis protocol at 8 months post-infection. **d** Serum HBsAg levels in HBV+DEN- (n = 4) and HBV+PBS-treated (n = 7) WT mice at 8 months after hydrodynamic injection of pAAV-HBV plasmid. Each dot represents a mouse. **e** HBV DNA levels in the serum of mice treated with HBV+DEN (n = 10) and HBV+PBS (n = 4) at 4 months post-hydrodynamic pAAV-HBV1.2 injection. HBV DNA positive and negative control samples are provided in the detection kit. Each dot represents a mouse. **f** Representative images of hepatitis B core antigen (HBcAg) stained liver tissues from HBV+DEN-, HBV+PBS-, Sham+DEN-, and Sham+PBS-treated mice at 4 months post-hydrodynamic pAAV-HBV1.2 injection. **g** Quantification of $HBcAg^{+}$ cells per high power field (HPF) in WT mice at 4 months after the hydrodynamic injection of pAAV-HBV plasmid. $HBcAg^{+}$ cells were counted in 5 to 10 randomly selected HPFs per liver. n = 4 mice in HBV+DEN group, n = 3 mice in Sham+DEN group, n = 3 mice in HBV+PBS group, and n = 3 mice in Sham+PBS group. Each dot represents one HPF. Graphs show mean + SD, **b**, **g**: one-way ANOVA with Tukey's multiple comparison test, **d**: two-sided unpaired *t*-test, scale bar: 100 μm. Source data are provided as a Source Data file.

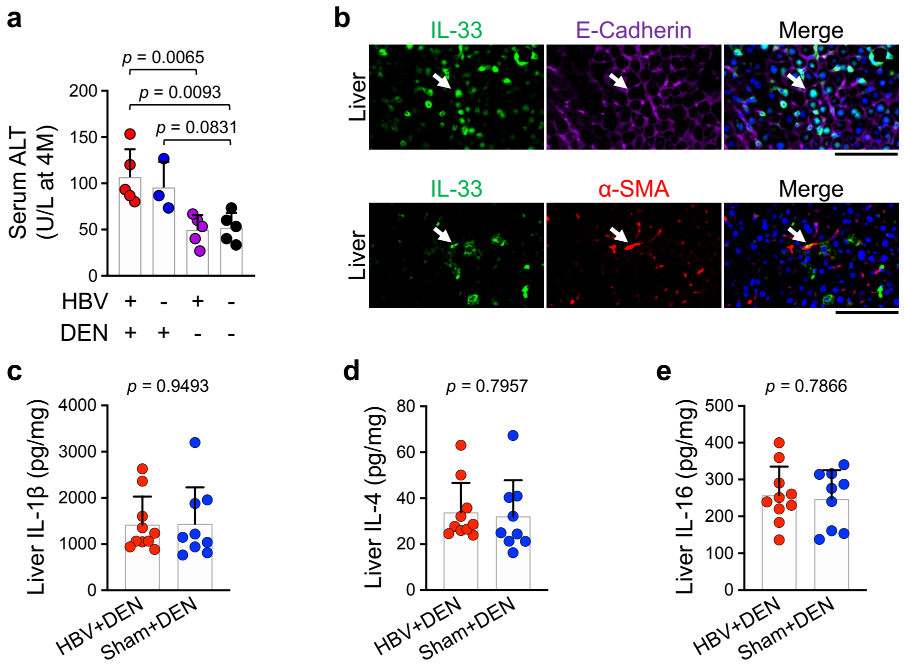

Supplementary Fig. 2, related to Fig. 2.

IL-33 cytokine is upregulated upon liver inflammation induced by HBV plus carcinogen treatment. a Quantification of serum ALT levels in WT mice that underwent liver carcinogenesis protocol at 4 months post-infection. n = 5 mice in HBV+DEN group, n = 3 mice in Sham+DEN group, n = 5 mice in HBV+PBS group, and n = 5 mice in Sham+PBS group. Each dot represents a mouse. **b** (top) Representative image of IL-33 and E-cadherin stained liver treated with carcinogenesis protocol at 4 months post-infection. The arrow points to an IL-33-expressing hepatocyte. (bottom) Representative image of IL-33 and α-SMA stained liver treated with carcinogenesis protocol at 4 months post-infection. The arrow points to an IL-33-expressing hepatic stellate cell. **c** IL-1β protein levels in HBV+DEN- (n = 10 mice) versus Sham+DEN-treated liver (n = 9 mice) at 4 months post-infection. Each dot represents a mouse. **d** IL-4 protein levels in HBV+DEN- (n = 10 mice) versus Sham+DEN-treated liver (n = 9 mice) at 4 months post-infection. Each dot represents a mouse. **e** IL-16 protein levels in HBV+DEN- (n = 10 mice) versus Sham+DEN-treated liver (n = 9 mice) at 4 months post-infection. Each dot represents a mouse.

Graphs show mean + SD, **a**: one-way ANOVA with Tukey's multiple comparison test, **c**, **d**, **e**: two-sided unpaired *t*-test. Source data are provided as a Source Data file.

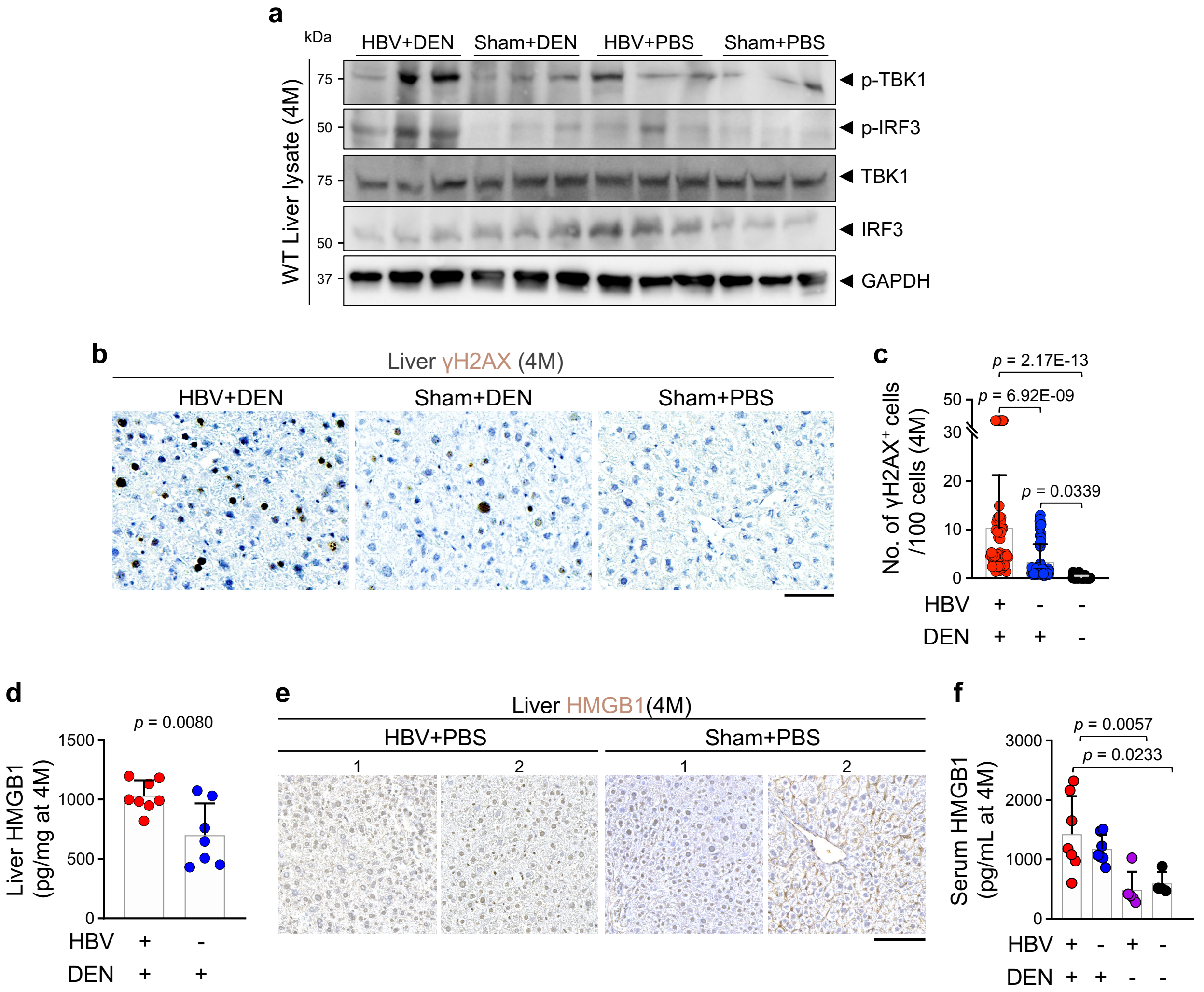

Supplementary Fig. 3, related to Fig. 2.

Hepatocytes experience increased DNA damage and immunogenic cell death after HBV plus carcinogen treatment. a Immunoblot of p-TBK1, p-IRF3, TBK1, IRF3, and GAPDH proteins in WT liver that received liver carcinogenesis protocol at 4 months post-infection (n = 3 mice in each group). The samples were derived from the same experiment but different gels for p-TBK1, another for p-IRF3, another for TBK1, another for IRF3, and another for GAPDH were processed in parallel. **b** Representative images of γH2AX stained HBV+DEN-, Sham+DEN-, and Sham+PBS-treated liver at 4 months post-infection. **c** Quantification of γH2AX^{+} cells per 100 cells in the HBV+DEN (n = 7 mice) compared with Sham+DEN- (n = 8 mice) and Sham+PBS-treated

liver (n = 6 mice) at 4 months post-infection. γH2AX^{+} cells per 100 cells were counted in five to ten randomly selected HPF images per liver. Each dot represents an HPF image. **d** HMGB1 levels in the liver of HBV+DEN- (n = 8) versus Sham+DEN-treated (n = 7) WT mice at 4 months post-infection. Each dot represents a mouse. **e** Representative images of HMGB1 stained HBV+PBS- and Sham+PBS-treated liver at 4 months post-infection. **f** Serum HMGB1 levels in WT mice that underwent liver carcinogenesis protocol at 4 months post-infection. n = 7 mice in HBV+DEN group, n = 7 mice in Sham+DEN group, n = 5 mice in HBV+PBS group, and n = 4 mice in Sham+PBS group. Each dot represents a mouse. Graphs show mean + SD, **c** and **f**: one-way ANOVA with Tukey's multiple comparison test, **d**: two-sided unpaired *t*-test, scale bars: 100 μm. Source data are provided as a Source Data file.

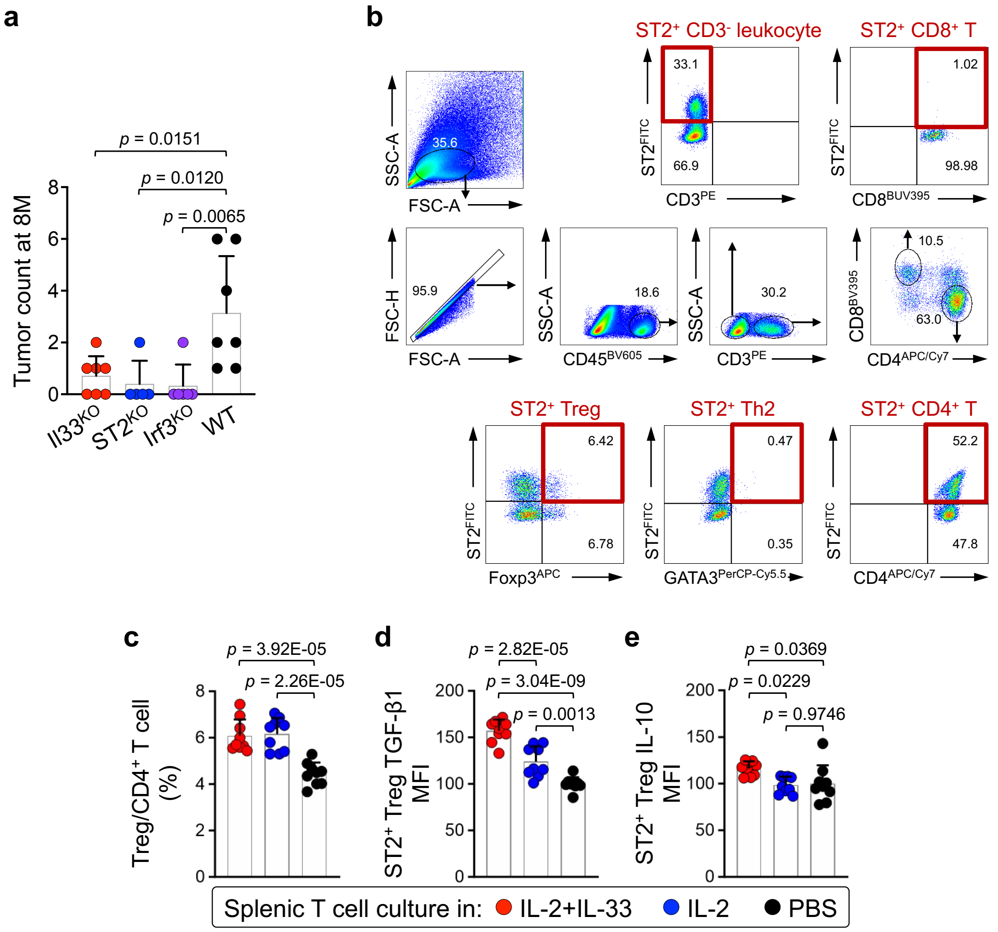

Supplementary Fig. 4, related to Fig. 3.

Tumor counts, flow gating strategy, and cytokine expression in splenic Tregs. a Liver tumor counts in Il33^{KO} (n = 7), ST2^{KO} (n = 5), Irf3^{KO} (n = 6), and WT (n = 7) mice that received HBV+DEN at 8 months post-infection. Each dot represents a mouse. **b** Flow gating strategy for immune cell types in mouse tissues (refer to Fig. 3e, 3f, and Supplementary Fig. 4c). Numbers on the flow plots represent % cells in the gate. **c** Splenic Treg frequency as % total CD4^{+} T cells in WT spleen after incubation with IL-2 plus IL-33, IL-2, versus no cytokine treatment (PBS) control (n = 9 culture wells per condition). Each dot represents a culture well. **d** TGF-β1 MFI of IL-2 plus IL-33, IL-2, versus PBS-treated ST2^{+} Tregs (n = 9 culture wells per condition). Each dot represents a culture well. **e** IL-10 MFI of IL-2 plus IL-33, IL-2, versus PBS-treated ST2^{+} Tregs (n = 9 culture wells per

condition). Each dot represents a culture well. Graphs show mean + SD, **a**, **c, d, e**: one-way ANOVA with Tukey's multiple comparison test. Source data are provided as a Source Data file.

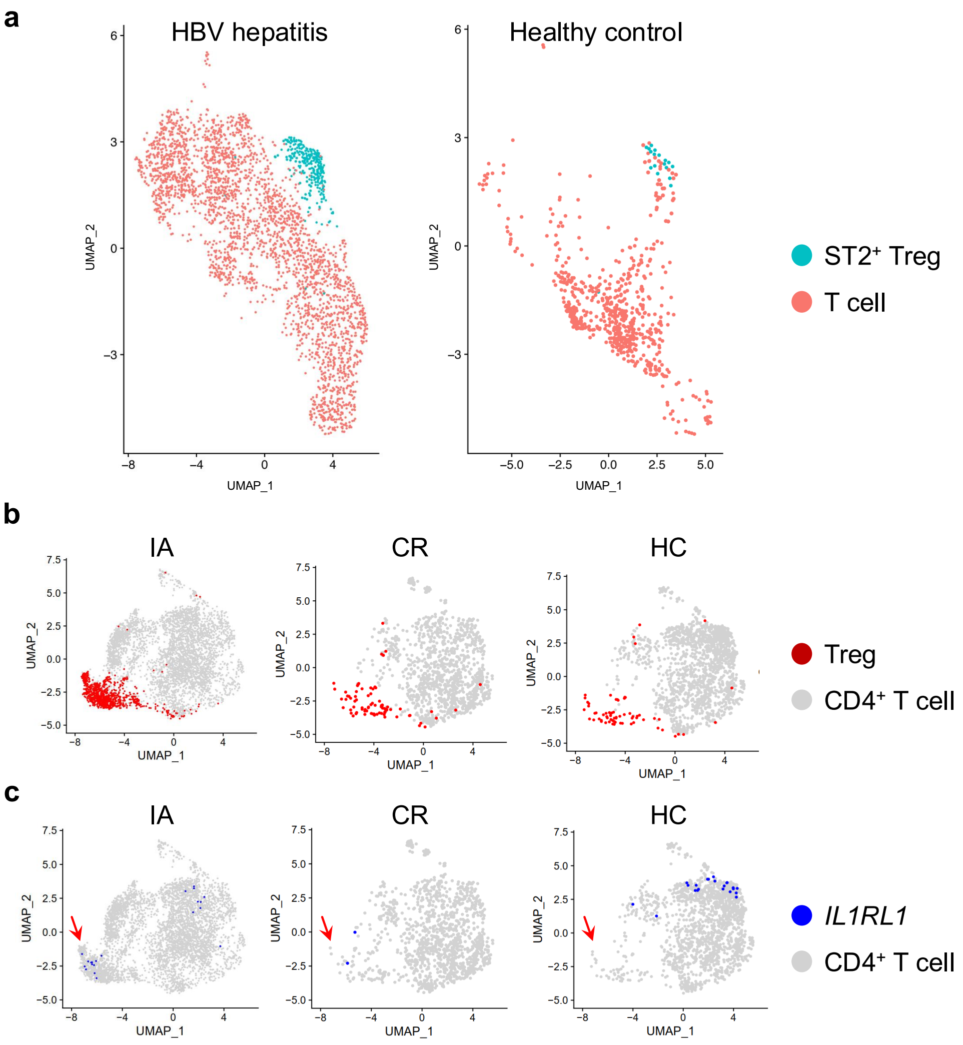

Supplementary Fig. 5, related to Fig. 3.

ST2$^+$ Tregs are increased in HBV-associated chronic hepatitis in humans. a scRNA-seq UMAP plot of ST2$^+$ Tregs (cyan dots) among T cells in human liver tissues from HBV-associated chronic hepatitis (n = 13) and healthy controls with no viral markers (n = 3). There are 175 ST2$^+$ Tregs out of 16021 T cells in HBV hepatitis (1.1%) and 18 ST2$^+$ Treg cells out of 2178 T cells in healthy control (0.8%). **b** scRNA-seq UMAP plots of Tregs (red dots) among CD4$^+$ T cells in human liver tissues from HBV-associated hepatitis (immune active or IA, n = 5), chronic resolved hepatitis (CR, HBV DNA level is below the detection limit, n = 3), and HBV-free healthy controls

(HC, n = 6). **c** *IL1RL1* expression highlights ST2$^+$ CD4$^+$ T cells (blue dots). The arrow points to the Treg cluster.

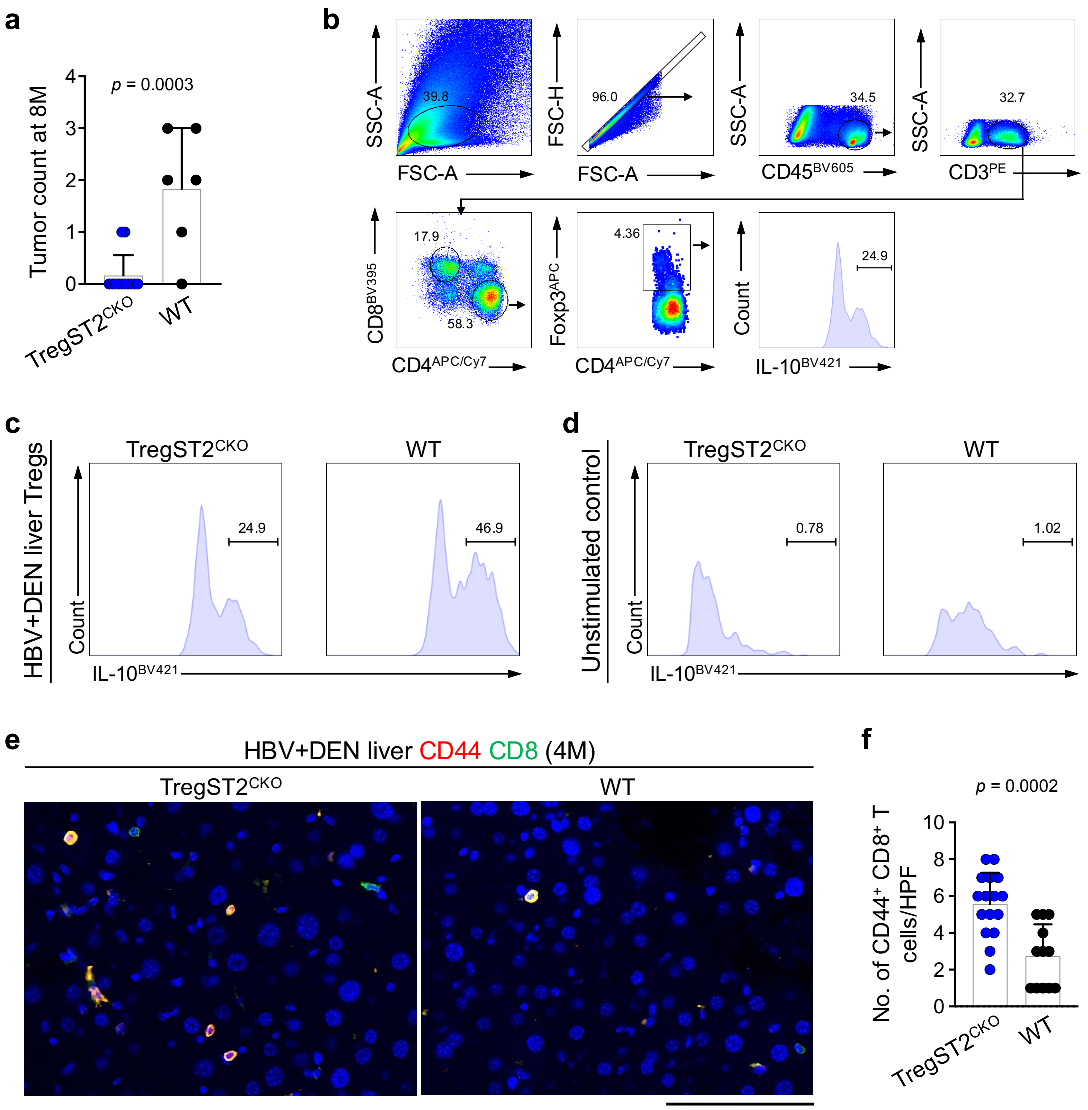

Supplementary Fig. 6, related to Fig. 4.

IL-10-expressing Tregs are reduced while activated CD8^{+} T cells are increased in TregST2^{CKO} liver after HBV plus carcinogen treatment. **a** Liver tumor counts in TregST2^{CKO} (n = 12) and WT mice (n = 6) that received HBV+DEN at 8 months post-infection. Each dot represents a mouse. **b** Flow gating strategy to identify IL-10-expressing Treg population in the liver (refer to Fig. 4f and Supplementary Fig. 6c, d). **c** Representative histograms of Tregs' IL-10 expression in HBV+DEN-treated TregST2^{CKO} and WT liver at 4 months post-infection. **d** Representative histograms of Tregs' IL-10 expression in samples not treated with cell activator

cocktail (unstimulated control) from HBV+DEN-treated TregST2^{CKO} and WT liver at 4 months post-infection. **e** Representative images of CD44 and CD8 stained TregST2^{CKO} and WT liver treated with HBV+DEN at 4 months post-infection. **f** Quantification of CD44^{+}CD8^{+} T cells in TregST2^{CKO} (n = 4) and WT (n = 3) liver treated with HBV+DEN at 4 months post-infection. Note that all CD8^{+} cells are CD3^{+} T cells. T cells were counted in four randomly selected HPF images per liver sample. Each dot represents an HPF image. Graphs show mean + SD, two-sided unpaired *t*-test, scale bar: 100 μm. Source data are provided as a Source Data file.

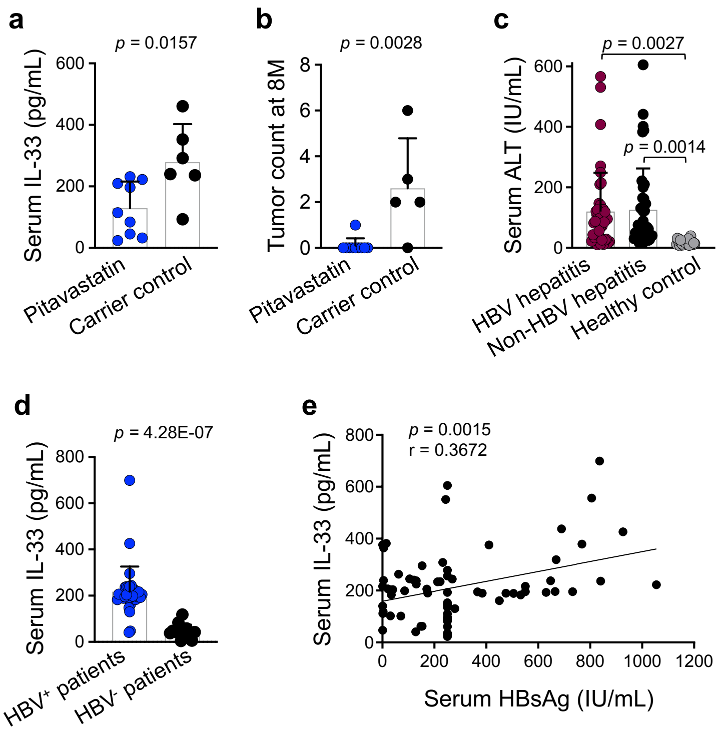

Supplementary Fig. 7, related to Fig. 5.

IL-33 levels in HBV^{+} and HBV^{-} patients. a Serum IL-33 levels in pitavastatin- (n = 9) and PBS-treated (n = 6) WT mice that received HBV+DEN at 8 months post-infection. Each dot represents a mouse. **b** Liver tumor counts in pitavastatin- (n = 10) and PBS-treated (n = 5) WT mice that received HBV+DEN at 8 months post-infection. Each dot represents a mouse. **c** Serum ALT levels in HBV^{+} hepatitis patients (n = 40), HBV^{-} hepatitis patients (n = 40), and healthy controls (n = 24). Each dot represents a patient serum sample. **d** Serum IL-33 levels in HBV^{+} (n = 32) and HBV^{-} patients (n = 15) with liver diseases (including HCC, benign tumor, hepatic cyst, and liver lipoma). Each dot represents a patient serum sample. **e** The correlation between serum IL-33 and HBsAg levels in HBV^{+} patients (n = 72) with liver diseases, including hepatitis, HCC, benign tumors, hepatic cysts, and liver lipoma. Each dot represents a patient. Graphs show mean + SD, **a**, **b, d**: two-sided unpaired *t*-test, **c**: one-way ANOVA with Tukey's multiple comparison test, **e**: two-sided *t*-test for Pearson correlation coefficient. Source data are provided as a Source Data file.

Supplementary Tables

Supplementary Table 1. Characteristics of pitavastatin and ezetimibe treatment cohorts.

**Baseline Characteristics: Matched Cohorts**				
	Pitavastatin		Ezetimibe	
Number of patients	242,645		242,645	
Age at index (years)	Mean	SD	Mean	SD
	63.8	10.9	63.4	11.7
Gender	N	%	N	%
Male	102,968	42.436	101,989	42.032
Female	139,588	57.528	140,591	57.941
Unknown	89	0.037	65	0.027
Race	Mean	%	Mean	%
White	79,571	32.793	76,501	31.528
Black	6,103	2.515	5,829	2.402
Asian	1,624	0.669	876	0.361
American Indian or	0	0	0	0
Native Hawaiian and	0	0	0	0
Unknown	155,347	64.022	159,439	65.709
Ethnicity	Mean	%	Mean	%
Hispanic or Latino	4,433	1.827	4,500	1.855
Not Hispanic or Latino	84,913	34.995	80,960	33.366
Unknown	153,299	63.178	157,185	64.78
