# Supplementary material for: Hepatitis B virus promotes liver cancer by modulating the immune response to environmental carcinogens
Source: Nat Commun. 2025 Jun 27;16:5360. doi: 10.1038/s41467-025-60894-z (PMC12205058; doi:10.1038/s41467-025-60894-z)
Supplement: Supplementary file 2 — Description of Additional Supplementary Information [file 41467_2025_60894_MOESM2_ESM.pdf]

Description of Supplementary Data Files

File Name: Supplementary Data 1

Description: Characteristics of patients and healthy controls. Peripheral blood samples from HBV hepatitis (A), Non-HBV hepatitis (including NASH, HCV, autoimmune hepatitis, etc.) (B), healthy controls (C), HBV^+ patients with other liver disease (D), and HBV^- patients with other liver disease (E) were obtained from the First Affiliated Hospital of the University of Science and Technology of China. The clinical characteristics of these patients and healthy controls are summarized. HBV^+ patients must have serum HBsAg test showing HBsAg > 0.08 IU/mL. HBV hepatitis group: n = 40, median age = 58 years (range 38-79), 7/40 (17.5%) female, and 33/40 (82.5%) male; Non-HBV hepatitis group: n = 40, median age = 57 years (range 12-78), 21/40 (52.5%) female, and 19/40 (47.5%) male; HBV^+ patients with other liver diseases group: n = 32, median age = 56.5 years (range 42-77), 9/32 (28.1%) female, and 23/32 (71.9%) male; HBV^- patients with other liver diseases group: n = 15, median age = 50 years (range 30-76), 7/15 (47%) female, and 8/15 (53%) male; healthy control group: n = 24, median age = 58.5 years (range 21-88), 13/24 (54.2%) female, and 11/24 (45.8%) male.

File Name: Supplementary Data 2

Description: Antibodies for western blotting, immunohistochemical and immunofluorescent staining, and flow cytometry.

File Name: Supplementary Data 3

Description: PCR primers for mouse genotyping.
