# Supplementary material for: Hepatitis B virus promotes liver cancer by modulating the immune response to environmental carcinogens
Source: Nat Commun. 2025 Jun 27;16:5360. doi: 10.1038/s41467-025-60894-z (PMC12205058; doi:10.1038/s41467-025-60894-z)
Supplement: Supplementary file 6 — Reporting Summary [file 41467_2025_60894_MOESM6_ESM.pdf]

nature portfolio

Corresponding author(s): Shadmehr Demehri
Last updated by author(s): Jun 5, 2025

Reporting Summary

Nature Portfolio wishes to improve the reproducibility of the work that we publish. This form provides structure for consistency and transparency in reporting. For further information on Nature Portfolio policies, see our Editorial Policies and the Editorial Policy Checklist.

Statistics

For all statistical analyses, confirm that the following items are present in the figure legend, table legend, main text, or Methods section.

n/a	Confirmed	
☐	☒	The exact sample size (n) for each experimental group/condition, given as a discrete number and unit of measurement
☐	☒	A statement on whether measurements were taken from distinct samples or whether the same sample was measured repeatedly
☐	☒	The statistical test(s) used AND whether they are one- or two-sided<br>*Only common tests should be described solely by name; describe more complex techniques in the Methods section.*
☒	☐	A description of all covariates tested
☐	☒	A description of any assumptions or corrections, such as tests of normality and adjustment for multiple comparisons
☐	☒	A full description of the statistical parameters including central tendency (e.g. means) or other basic estimates (e.g. regression coefficient) AND variation (e.g. standard deviation) or associated estimates of uncertainty (e.g. confidence intervals)
☐	☒	For null hypothesis testing, the test statistic (e.g. F, t, r) with confidence intervals, effect sizes, degrees of freedom and P value noted<br>*Give P values as exact values whenever suitable.*
☒	☐	For Bayesian analysis, information on the choice of priors and Markov chain Monte Carlo settings
☒	☐	For hierarchical and complex designs, identification of the appropriate level for tests and full reporting of outcomes
☐	☒	Estimates of effect sizes (e.g. Cohen's d, Pearson's r), indicating how they were calculated

Our web collection on statistics for biologists contains articles on many of the points above.

Software and code

Policy information about availability of computer code

Data collection	FlowJo™ v10 Software : For flow cytometry data<br><br>Zeiss Zen software (2014 S4): For immunofluorescence images<br>NDP.View2: For histology images<br><br>cSeries Capture software: For all the WB images and calculation of band intensity.<br>Envision manager 1.14.3049.528: For checking absorbance of protein<br><br>Gen 5 2.09: For checking ELISA data collection.<br><br>Illumina Real Time Analysis software (RTA1.13): For RNA-Seq<br><br>R(4.2.3): For scRNA-seq
Data analysis	Graphpad Prism 10 (to confirm significancy and values)

For manuscripts utilizing custom algorithms or software that are central to the research but not yet described in published literature, software must be made available to editors and reviewers. We strongly encourage code deposition in a community repository (e.g. GitHub). See the Nature Portfolio guidelines for submitting code & software for further information.

Data

Policy information about availability of data

All manuscripts must include a data availability statement. This statement should provide the following information, where applicable:

- Accession codes, unique identifiers, or web links for publicly available datasets
- A description of any restrictions on data availability
- For clinical datasets or third party data, please ensure that the statement adheres to our policy

All raw needed to evaluate the conclusions in the paper are present in the paper and/or the supplementary information. RNA sequencing data can be accessed from the NCBI gene expression omnibus (GEO), accession no: GSE269528. scRNA sequencing data can be accessed from GEO dataset, accession no: GSE234241, GSE182159.

All gel images from western blot and source data are provided with this paper.

Research involving human participants, their data, or biological material

Policy information about studies with human participants or human data. See also policy information about sex, gender (identity/presentation), and sexual orientation and race, ethnicity and racism.

Reporting on sex and gender	No sex or gender were considered in the study design.
Reporting on race, ethnicity, or other socially relevant groupings	No race, ethnicity or other socially relevant groupings were considered in the study design.
Population characteristics	Peripheral blood samples from HBV hepatitis, Non-HBV hepatitis (including NASH, HCV, autoimmune hepatitis, etc.), healthy controls, HBV+ patients with other liver disease, and HBV- patients with other liver disease were obtained from the First Affiliated Hospital of the University of Science and Technology of China. HBV+ patients must have serum HBsAg test with HBsAg > 0.08 IU/mL. HBV hepatitis group: n = 40, median age = 58 years (range 38-79), 7/40 (17.5%) female, and 33/40 (82.5%) male; Non-HBV hepatitis group: n = 40, median age = 57 years (range 12-78), 21/40 (52.5%) female, and 19/40 (47.5%) male; HBV+ patients with other liver diseases group: n = 32, median age = 56.5 years (range 42-77), 9/32 (28.1%) female, and 23/32 (71.9%) male; HBV- patients with other liver diseases group: n = 15, median age = 50 years (range 30-76), 7/15 (47%) female, and 8/15 (53%) male; healthy control group: n = 24, median age = 58.5 years (range 21-88), 13/24 (54.2%) female, and 11/24 (45.8%) male. More detailed patients demographics information is listed in supplemental Data 1.
Recruitment	The study recruited HBV+ and HBV- hepatitis, and other liver diseases (HCC, cirrhosis, hepatic cyst, fatty liver, liver hemangioma)patients at the First Affiliated Hospital of USTC. HBV+ patients must have serum HBsAg test with HBsAg>0.08 IU/mL. Serum and liver biopsy were collected for protein analysis. All patients provided written informed consent before the study.
Ethics oversight	The study was conducted in accordance with the ethical principles described in the Declaration of Helsinki, and approved by the Ethics Committees of the First Affiliated Hospital of University of Science and Technology of China (USTC) (2020-KYLS-124)

Note that full information on the approval of the study protocol must also be provided in the manuscript.

Field-specific reporting

Please select the one below that is the best fit for your research. If you are not sure, read the appropriate sections before making your selection.

☒ Life sciences ☐ Behavioural & social sciences ☐ Ecological, evolutionary & environmental sciences

For a reference copy of the document with all sections, see nature.com/documents/nr-reporting-summary-flat.pdf

Life sciences study design

All studies must disclose on these points even when the disclosure is negative.

Sample size	Sample sizes were not predetermined based on statistical methods, but were chosen accroding to the standards of the field (at least three independent biological replicates for each condition), which generated a sufficient number of single-molecule trajectories and gave sufficient statistics for the effect sizes of interest and similar to our previous experience from our group (PMID: 33616251, 38816352)<br><br>We utilized at least three times independent in vitro study and 3-10 mice/group in this study.
Data exclusions	we did not exclude any data from consideration
Replication	Reported results were consistently replicated across multiple experiments with all replicates generating similar results.

Randomization	All mice were randomly assigned to the control or experimental groups.
Blinding	Investigators were not blinded. Blinding during collection was not needed because conditions were well controlled. Blinding during analysis was not feasible as the differences between samples under different conditions were visually apparent in the trajectories. Blinding is also not nessasary because the results were quantitative and did not require subjective judgment or interpretation. Blinding is not typically used in the field.

Behavioural & social sciences study design

All studies must disclose on these points even when the disclosure is negative.

Study description	N/A
Research sample	N/A
Sampling strategy	N/A
Data collection	N/A
Timing	N/A
Data exclusions	N/A
Non-participation	N/A
Randomization	N/A

Ecological, evolutionary & environmental sciences study design

All studies must disclose on these points even when the disclosure is negative.

Study description	N/A
Research sample	N/A
Sampling strategy	N/A
Data collection	N/A
Timing and spatial scale	N/A
Data exclusions	N/A
Reproducibility	N/A
Randomization	N/A
Blinding	N/A

Did the study involve field work? ☐ Yes ☒ No

Field work, collection and transport

Field conditions	N/A
Location	N/A
Access & import/export	N/A
Disturbance	N/A

Reporting for specific materials, systems and methods

We require information from authors about some types of materials, experimental systems and methods used in many studies. Here, indicate whether each material, system or method listed is relevant to your study. If you are not sure if a list item applies to your research, read the appropriate section before selecting a response.

Materials & experimental systems

n/a	Involved in the study
☐	☒ Antibodies
☒	☐ Eukaryotic cell lines
☒	☐ Palaeontology and archaeology
☐	☒ Animals and other organisms
☒	☐ Clinical data
☒	☐ Dual use research of concern
☒	☐ Plants

Methods

n/a	Involved in the study
☒	☐ ChIP-seq
☐	☒ Flow cytometry
☒	☐ MRI-based neuroimaging

Antibodies

Antibodies used

For western blot,
1st antibodies:
p-TBK1 (Cell signaling technologies, #5483, D52C2), used at 1:1000
p-IRF3 (Cell signlaing technologies, #29047, D6O1M), used at 1:1000
TBK1 (Cell signaling technologies, #3504, D1B4), used at 1:1000
IRF3 (Cell signaling technologies, #4302, D83B9), used at 1:1000
GAPDH (Cell signaling technologies, #5174, D16H11), used at 1:1000
2nd antibodies:
Peroxidase Goat anti-rabbit IgG (Jackson immunoresearch, 111-035-003, Poly-clonal), used at 1:5000

For Histology,
1st antibodies:
PCNA(Cell signaling technology, #2586, PC10), used at 1:5000
HMGB1(Cell signaling technology, #6893, D3E5), used at 1:200
Mouse IL-33 (Enzo, #ALX804840C100, Nessy-1), used at 1:200
Human IL-33 (Sigma, #HPA024426, Poly=clonal), used at 1:200
CD45 (Abcam, #AB10558, Bra55), used at 1:500
CD3 (Abcam, #AB11089, CD3-12), used at 1:500
CD8 (Cell signaling technology, #98941, D4W2Z), used at 1:500
CD44 (Cell signaling technology, #37259, E7K2Y), used at 1:200
rH2AX (Cell signaling technology, #9718, 20E3), used at 1:500
E-cadherin (BD science, #610181, 36/E-cadherin (RUO)), used at 1:200
aSMA (Cell signaling technology, #19245, D4K9N), used at 1:100
HBcAg (Abcam, #ab316283, EPR28251-34), used at 1:800

2nd antibodies:
Goat anti-Rabbit IgG, Alexa Fluor 488 conjugate (Thermo Fisher Scientific, #11034, Poly-clonal), used at 1:200
Goat anti-Rabbit IgG, Alexa Fluor 568 conjugate (Thermo Fisher Scientific, #11036, Poly-clonal), used at 1:200
Goat anti-mouse IgG(H+L), AF488 (Southern Biotech, #1031-30, Poly-clonal), used at 1:200
Goat anti-Rat IgG(H+L), Cross-adsorbed secondary antibody, Alexa Fluor 647 (Thermo Fisher scientific, A-21247, Poly-clonal), used at 1:200
Goat anti-Rabbit IgG, Alexa Fluor 635 conjugate (Thermo Fisher scientific, #A31577, Poly-clonal), used at 1:200
Goat anti-Rabbit IgG, (BD bioscience, #550338, Poly-clonal), used at 1:200
Goat anti-Mouse IgG, (Biolegend, #405303, Poly-clonal), used at 1:200

For flow cytometric analysis,
BV605-CD45 (Biolegend, #103139, 30-F11), used at 1:200
PE-CD3 (Biolegend, #100307, 145-2C11), used at 1:200
eFlour450-CD3 (eBioscience, #16-0031-82, 145-2C11), used at 1:200
APC/Cy7-CD4 (Biolegend, #100525, RM4-5), used at 1:200
BUV395-CD8 (BD biosciences, #565968, 53-6.7), used at 1:200
FITC-ST2 (MDBIOPRODUCTS, #101001F, DJ8), used at 1:200
AF700-CD25 (Biolegned, #102024, PC61), used at 1:200
PerCP/Cy5.5-GATA3 (Biolegend, #653811, 16E10A23), used at 1:100
APC-FoxP3 (eBioscience, #17-5773-82, FJK-16s), used at 1:100
BV421-IL10 (BD Bioscience, #563276, JES5-16E3), used at 1:100

PerCP/Cy5.5 TGFbeta1 (Biolegend, #141409, TW7-16B4), used at 1:100

Validation

p-TBK1 (CST, 5483)
https://www.cellsignal.com/products/primary-antibodies/phospho-tbk1-nak-ser172-d52c2-xp-rabbit-mab/5483?srsltid=AfmBOormlZrQDmZ7UrnhOISE1nFTzQW0TYf_UYKbWSP2gP_tb94UpDL2
p-IRF3 (CST, 29047)
https://www.cellsignal.com/products/primary-antibodies/phospho-irf-3-ser396-d6o1m-rabbit-mab/29047
TBK1 (CST, 3504)
https://www.cellsignal.com/products/primary-antibodies/tbk1-nak-d1b4-rabbit-mab/3504
IRF3 (CST, 4302)
https://www.cellsignal.com/products/primary-antibodies/irf-3-d83b9-rabbit-mab/4302
GAPDH (CST, 5174)
https://www.cellsignal.com/products/primary-antibodies/gapdh-d16h11-xp-rabbit-mab/5174
Peroxidase Goat anti-rabbit IgG (111-035-003)
https://www.jacksonimmuno.com/catalog/products/111-035-003

PCNA(CST, 2586)
https://www.cellsignal.com/products/primary-antibodies/pcna-pc10-mouse-mab/2586?srsltid=AfmBOooWK9bsz5hVvjzIc0a1-qNkmc9-NvVtzCQPpW8iFXkSELkGs6UT
HMGB1 (CST,6893)
https://www.cellsignal.com/products/primary-antibodies/hmgb1-d3e5-rabbit-mab/6893
Mouse IL-33 (Enzo, Nessy-1)
https://www.enzo.com/product/il-33-monoclonal-antibody-nessy-1/
Human IL-33 (Sigma, polyclonal)
https://www.sigmaaldrich.com/KR/ko/product/sigma/hpa024426?srsltid=AfmBOoqHb8ZND494P4bZdPnJasIHz1FioAeb7FEORfus7Ug6TxgsUCVf
CD45 (Abcam, Bra55)
https://www.abcam.com/en-us/products/primary-antibodies/cd45-antibody-ab10558
CD3 (Abcam, CD3-12)
https://www.abcam.com/en-us/search?productSorting=relevance&resourceSorting=relevance&keywords=11089
CD8 (CST, D4W2Z)
https://www.cellsignal.com/products/primary-antibodies/cd8a-d4w2z-xp-rabbit-mab/98941
CD44 (CST, E7K2Y)
https://www.cellsignal.com/products/primary-antibodies/cd44-e7k2y-xp-rabbit-mab/37259?srsltid=AfmBOoqtgHQdL60bXX1qRZMdW7ffbbg6Vw1we1frCv88zw3uXSaYmPNT
rH2AX (CST, 20E3)
https://www.cellsignal.com/products/primary-antibodies/phospho-histone-h2a-x-ser139-20e3-rabbit-mab/9718
E-Cadherin (BD, 36/E-Cadherin(RUO))
https://www.bdbiosciences.com/ko-kr/products/reagents/microscopy-imaging-reagents/immunofluorescence-reagents/purified-mouse-anti-e-cadherin.610181?tab=product_details
aSMA (CST, D4K9N)
https://www.cellsignal.com/products/primary-antibodies/a-smooth-muscle-actin-d4k9n-xp-rabbit-mab/19245
HBcAg (EPR28251-34)
https://www.abcam.com/en-us/products/primary-antibodies/hepatitis-b-virus-core-antigen-antibody-epr28251-34-ab316283?srsltid=AfmBOopZSuSlnkExznakvBJPjO_LhYggWRevIyON1-ydgMHjQJfAllW

Rabbit, AF488 (Thermo fisher, 11034)
https://www.thermofisher.com/antibody/product/Goat-anti-Rabbit-IgG-H-L-Highly-Cross-Adsorbed-Secondary-Antibody-Polyclonal/A-11034
Goat anti-Rabbit AF568 (Thermo fisher scientific, 11036)
https://www.thermofisher.com/antibody/product/Goat-anti-Rabbit-IgG-H-L-Highly-Cross-Adsorbed-Secondary-Antibody-Polyclonal/A-11036
Goat anti-mouse AF488 (Southern Biotech, #1031-30)
https://www.southernbiotech.com/goat-anti-mouse-igg-h-l-human-ads-af488-1031-30
Goat anti-Rat AF647 (Invitrogen, a-21247)
https://www.thermofisher.com/antibody/product/Goat-anti-Rat-IgG-H-L-Cross-Adsorbed-Secondary-Antibody-Polyclonal/A-21247

BV605-CD45 (Biolegend, 30-F11)
https://www.biolegend.com/fr-lu/products/brilliant-violet-605-anti-mouse-cd45-antibody-8721
PE-CD3 (Biolegend, 145-2C11)
https://www.biolegend.com/nl-be/products/pe-anti-mouse-cd3epsilon-antibody-25
eFlour450-CD3 (eBioscience, 145-2C11)
https://www.thermofisher.com/antibody/product/CD3e-Antibody-clone-145-2C11-Monoclonal/16-0031-82

APC/Cy7-CD4 (Biolegend, RM4-5)
https://www.thermofisher.com/antibody/product/CD3e-Antibody-clone-145-2C11-Monoclonal/16-0031-82
BUV395-CD8 (BD Bioscience, 53-6.7)
https://www.bdbiosciences.com/en-us/products/reagents/flow-cytometry-reagents/research-reagents/single-color-antibodies-ruo/buv395-rat-anti-mouse-cd8a.565968?tab=product_details
FITC-ST2 (MDBIOPRODUCTS, DJ8)
https://www.mdbioproducts.com/products/t1-st2-il-33r-mouse-monoclonal-antibody-fitc?variant=39848199848125
AF700 (Biolegend, PC61)
https://www.biolegend.com/de-de/products/alexa-fluor-700-anti-mouse-cd25-antibody-3389
PerCP/Cy5.5-GATA3 (Biolegend, 16E10A23)
https://www.biolegend.com/fr-fr/products/percp-cyanine5-5-anti-gata3-antibody-9400?GroupID=GROUP26
APC-FoxP3 (eBioscience, FJK-16s)
https://www.thermofisher.com/antibody/product/FOXP3-Antibody-clone-FJK-16s-Monoclonal/17-5773-82
BV421-IL10 (BD Bioscience, JES5-16E3)
https://www.bdbiosciences.com/ko-kr/products/reagents/flow-cytometry-reagents/research-reagents/single-color-antibodies-ruo/bv421-rat-anti-mouse-il-10.563276?tab=product_details
PerCP/Cy5.5 TGFbeta1 (Biolegend, TW7-16B4)
https://www.biolegend.com/nl-be/products/percp-cyanine5-5-anti-mouse-lap-tgf-beta1-antibody-8193

Eukaryotic cell lines

Policy information about cell lines and Sex and Gender in Research

Cell line source(s)	N/A
Authentication	N/A
Mycoplasma contamination	N/A
Commonly misidentified lines (See ICLAC register)	N/A

Palaeontology and Archaeology

Specimen provenance	N/A
Specimen deposition	N/A
Dating methods	N/A

☐ Tick this box to confirm that the raw and calibrated dates are available in the paper or in Supplementary Information.

Ethics oversight	N/A

Note that full information on the approval of the study protocol must also be provided in the manuscript.

Animals and other research organisms

Policy information about studies involving animals; ARRIVE guidelines recommended for reporting animal research, and Sex and Gender in Research

Laboratory animals	Wild-type and mutant male mice on C57BL/6 background (4-6 weeks age) were used in the studies. For mutant mice, Irf3KO mice were purchased from the Riken Bioresource research center (Ibaraki, Japan). Il33KO mice were a gift from Dr. Marco Colonna, and ST2KO mice were from Dr. Peter Nigrovic. TregST2CKO mice were generated by Diane Mathis and Richard T. Lee. C57BL/6 WT mice were purchased from the jackson laboratory (Bar Harbor, ME). All mice were housed in an environment with a 12-hour light/dark cycle, maintaining an ambient temperature between 21-25C and 40-60% humidity levels.
Wild animals	No wild animals were used in this study.
Reporting on sex	Male mice were used in the studies, since it was reported that male mice are more susceptible for HBV infection establishment and cancer progression in this model.
Field-collected samples	No field-collected samples were used in this study
Ethics oversight	All mice used in the studies were maintained under specific pathogen-free conditions in an MGH CCM facility. The mouse work was performed under the study protocol #2015N000089, as approved by the institutional animal care and use committee. All mice used in the studies were euthanized under anesthesia.

Note that full information on the approval of the study protocol must also be provided in the manuscript.

Clinical data

Policy information about clinical studies
All manuscripts should comply with the ICMJEguidelines for publication of clinical research and a completedCONSORT checklist must be included with all submissions.

Clinical trial registration	N/A
Study protocol	N/A
Data collection	N/A
Outcomes	N/A

Dual use research of concern

Policy information about dual use research of concern

Hazards

Could the accidental, deliberate or reckless misuse of agents or technologies generated in the work, or the application of information presented in the manuscript, pose a threat to:

No	Yes	
x		Public health
x		National security
x		Crops and/or livestock
x		Ecosystems
x		Any other significant area

Experiments of concern

Does the work involve any of these experiments of concern:

No	Yes	
x		Demonstrate how to render a vaccine ineffective
x		Confer resistance to therapeutically useful antibiotics or antiviral agents
x		Enhance the virulence of a pathogen or render a nonpathogen virulent
x		Increase transmissibility of a pathogen
x		Alter the host range of a pathogen
x		Enable evasion of diagnostic/detection modalities
x		Enable the weaponization of a biological agent or toxin
x		Any other potentially harmful combination of experiments and agents

Plants

Seed stocks	N/A
Novel plant genotypes	N/A
Authentication	N/A

ChIP-seq

Data deposition

☐ Confirm that both raw and final processed data have been deposited in a public database such as GEO.

☐ Confirm that you have deposited or provided access to graph files (e.g. BED files) for the called peaks.

Data access links<br>*May remain private before publication.*	N/A
Files in database submission	N/A
Genome browser session<br>(e.g. UCSC)	N/A

Methodology

Replicates	N/A
Sequencing depth	N/A
Antibodies	N/A
Peak calling parameters	N/A
Data quality	N/A
Software	N/A

Flow Cytometry

Plots

Confirm that:

☒ The axis labels state the marker and fluorochrome used (e.g. CD4-FITC).

☒ The axis scales are clearly visible. Include numbers along axes only for bottom left plot of group (a 'group' is an analysis of identical markers).

☒ All plots are contour plots with outliers or pseudocolor plots.

☒ A numerical value for number of cells or percentage (with statistics) is provided.

Methodology

Sample preparation	Livers from mice were harvested, mashed through a 100 μm filter and centrifuged at 2000 g for 1 minute to remove hepatocytes. The samples were then centrifuged at 3000 g to collect cells. The pellets were resuspended in 40% isotonic Percoll (Merck), a mixture of Percoll with 1X PBS and 10X PBS, and the samples were gently layered onto 70% Percoll. After centrifugation at 3000 g with very low deceleration conditions, cells were collected from the interface between the 40% and 70% Percoll layers. The cells were washed with PBS and incubated in RPMI 1640 (Life Technologies) supplemented with 10% FBS (Corning) and 1% penicillin and streptomycin (Thermo Fisher Scientific) (R10 media) along with a cell activator cocktail (Biolegend) at 37°C. After one hour of incubation, monensin (Biolegend) and brefeldin A (Biolegend) were added, and the cells were incubated for an additional three hours at 37°C. The cells were stained with the following surface marker antibodies. They were then fixed and permeabilized using the True-Nuclear Transcription Factor Buffer Set (Biolegend) for staining with intracellular markers. The stained cells were analyzed using a LSR Fortessa X-20 flow cytometer (BD Bioscience)<br><br>Spleens from C57BL/6 mice were harvested and meshed through a 70 μm filter, and the cells were resuspended in RBC lysis buffer for 3 minutes. After washing with PBS, the cells were counted, and 5 x 10^6 splenocytes were resuspended in 1 mL of R10 media containing 2 μg/mL of CD28 antibody (BioXcell, Lebanon). The cells were then plated in a 12-well plate coated with 10 μg/mL of CD3 antibody (BioXcell) and incubated at 37°C. After 24 hours, PBS, IL-2 (20 ng/mL), or IL-2 (20 ng/mL) plus IL-33 (200 ng/mL) was added to the cells. The next day, monensin and brefeldin A were added and the samples were incubated for an additional four hours at 37°C. The stained cells were analyzed using a LSR Fortessa X-20 flow cytometer (BD Bioscience)
Instrument	The stained cells were analyzed using LSR Fortessa X-20 BD flow cytometer
Software	BD FACS DIVA and FlowJo software
Cell population abundance	At least 10,000 cells were analyzed for In vitro study. For in vivo, at least 50,000 cells were analyzed.

Gating strategy	Cells were gated according to SSC-A&FSC-A. Doublets were excluded by FSC-A&FSC-H. Gating strategies for specific cell population were shown in supplementary figures.

☒ Tick this box to confirm that a figure exemplifying the gating strategy is provided in the Supplementary Information.

Magnetic resonance imaging

Experimental design

Design type	N/A
Design specifications	N/A
Behavioral performance measures	N/A

Acquisition

Imaging type(s)	N/A
Field strength	N/A
Sequence & imaging parameters	N/A
Area of acquisition	N/A

Diffusion MRI ☐ Used ☒ Not used

Preprocessing

Preprocessing software	N/A
Normalization	N/A
Normalization template	*Describe the template used for normalization/transformation, specifying subject space or group standardized space (e.g. original Talairach, MNI305, ICBM152) OR indicate that the data were not normalized.*
Noise and artifact removal	*Describe your procedure(s) for artifact and structured noise removal, specifying motion parameters, tissue signals and physiological signals (heart rate, respiration).*
Volume censoring	N/A

Statistical modeling & inference

Model type and settings	N/A
Effect(s) tested	N/A

Specify type of analysis: ☐ Whole brain ☐ ROI-based ☐ Both

Statistic type for inference (See Eklund et al. 2016)	N/A
Correction	N/A

Models & analysis

n/a	Involved in the study
☒	☐ Functional and/or effective connectivity
☒	☐ Graph analysis
☒	☐ Multivariate modeling or predictive analysis

Functional and/or effective connectivity	*Report the measures of dependence used and the model details (e.g. Pearson correlation, partial correlation, mutual information).*
Graph analysis	*Report the dependent variable and connectivity measure, specifying weighted graph or binarized graph, subject- or group-level, and the global and/or node summaries used (e.g. clustering coefficient, efficiency, etc.).*

Multivariate modeling and predictive analysis	*Specify independent variables, features extraction and dimension reduction, model, training and evaluation metrics.*
